# Mesoporous TiO_2_/Carbon Beads: One-Pot Preparation and Their Application in Visible-Light-Induced Photodegradation

**DOI:** 10.1007/s40820-015-0029-5

**Published:** 2015-03-24

**Authors:** Xiaowei Li, Yanqiu Jiang, Wenjing Cheng, Yudong Li, Xianzhu Xu, Kaifeng Lin

**Affiliations:** Academy of Fundamental and Interdisciplinary Sciences, Harbin Institute of Technology, Harbin, 150080 People’s Republic of China

**Keywords:** TiO_2_ nanoparticles, Mesoporous carbon, Composite bead, Visible light, Easy separation

## Abstract

Mesoporous TiO_2_/Carbon beads have been prepared via a facile impregnation-carbonization approach, in which a porous anion-exchange resin and K_2_TiO(C_2_O_4_)_2_ were used as hard carbon and titanium source, respectively. Characterization results reveal that the self-assembled composites have disordered mesostructure, uniform mesopores, large pore volumes, and high surface areas. The mesopore walls are composed of amorphous carbon, well-dispersed and confined anatase or rutile nanoparticles. Some anatase phase of TiO_2_ was transformed to rutile phase via an increase of carbonization temperature or repeated impregnation of the resin with TiO(C_2_O_4_)_2_^2−^ species. X-ray photoelectron spectroscopy, carbon, hydrogen, and nitrogen element analysis, and thermal gravity analysis results indicate the doping of carbon into the TiO_2_ lattice and strong interaction between carbon and TiO_2_ nanoparticles. A synergy effect by carbon and TiO_2_ in the composites has been discussed herein on the degradation of methyl orange under visible light. The dye removal process involves adsorption of the dye from water by the mesopores in the composites, followed by photodegradation on the separated dye-loaded catalysts. Mesopores allow full access of the dye molecules to the surface of TiO_2_ nanoparticles. Importantly, the bead format of such composite enables their straightforward separation from the reaction mixture in their application as a liquid-phase heterogeneous photodegradation catalyst.

## Introduction

Among various inorganic nanomaterials, titania (TiO_2_) is regarded as the most promising photocatalysts in water splitting and the mineralization of toxic organic substances as its attractive features of low-cost, nontoxicity, and high stability [1–5]. For major applications, however, problems with the use of these TiO_2_ nanoparticles are also well recognized: (1) titania absorbs only ultraviolet light; (2) the loss of the nanoparticles in solution and aggregation of the nanoparticles in suspension. To improve the energy efficiency, several efforts have been made in developing new photocatalysts by modifying the TiO_2_ sheets with nonmetal elements to narrow the band gap and enhance activities under visible light (>420 nm) [6–14]. Carbon modifications including carbon doping, carbon-related coating, and carbon loading have been touted as one of ideal and inexpensive choices for narrowing the band gap and increasing the visible light response of TiO_2_ nanoparticles [15–18]. Despite the complexities in synthetic procedures, the carbon modified TiO_2_ nanoparticles have shown higher photocatalytic activity than the non-modified analog during the degradation of substances under visible light. Among the modifications, TiO_2_ nanoparticles loaded on carbons have been extensively investigated [19–22]. It has been proved that such composites have a large electron-storage capacity and can accept the photon-excited electrons to promote the separation of photo-generated carries. Besides, TiO_2_ in such materials could be sensitized by absorbing more visible light [23].

Soft and hard carbon sources have been used as the based materials and activated carbons to prepare the TiO_2_/carbon (TiO_2_/C) composites. For examples, Zhao et al. utilized furfural as the soft carbon source to synthesize carbon@TiO_2_ with visible-light activity by solvothermal synthesis procedure [24]. In the photodegradation progress, the direct optical charge transfer transition was completed by both the carbon and TiO_2_. Zhang et al. synthesized nano-sized TiO_2_-supported activated carbon and realized the microwave-induced degradation of parathion with coal powder carbon precursor [25]. In order to overcome the drawback of the loss of TiO_2_ nanoparticles in solution and aggregation of TiO_2_ nanoparticles in suspension, active carbon sources with certain shape have been applied for preparation of such composites; however, most of them were investigated under ultraviolet (UV) light. Asiltürk et al. employed chemical activated pine sawdust as activated carbon support for TiO_2_, and the materials exhibited high activities for the degradation of Rhodamine B under UV light [26]. Baek et al. used the spherical strong acid ion-exchange resin as carbon precursor to support TiO_2_ and degrade humic acid under UV irradiation [27]. Shi et al. utilized rayon as the activated carbon fiber precursor and synthesized TiO_2_/activated carbon fibers photocatalyst that degraded methylene blue under ultraviolet irradiation [28].

Inspired by the studies on the preparation of spherical carbon structures [29–31], herein, we report a one-pot approach for preparation of mesoporous TiO_2_/C beads obtained by a facile anion-exchange progress and the subsequent carbonization procedure. Under visible light, the obtained beads exhibited high photocatalytic activity. More importantly, the separation of the beads from the reaction solution was extremely straightforward and did not require any centrifugation or filtration because the bead format was not destroyed during photocatalysis. This work may realize the wide application of TiO_2_, and has a certain practical significance.

## Experimental

### Preparation of Photocatalysts

The incorporation of TiO_2_ nanoparticles into the activated carbon matrix was performed by a general impregnation-carbonization approach, in which Amberlite IRA-900 resin and potassium titanium oxide oxalate dihydrate (K_2_TiO(C_2_O_4_)_2_·2H_2_O) were used as carbon and titanium precursors, respectively. Amberlite IRA-900 resin is in the chloride form with bead size of 290–480 µm (measured by optical microscope and determined by more than 50 beads), and is a strongly basic, macroreticular resin with moderately high porosity and with benzyltrialkylammonium functionalities [32]. The titanium precursor was purchased from Sinopharm Chemical Reagent Co., Ltd and was used without purification.

In a typical synthesis, 0.9 g of K_2_TiO(C_2_O_4_)_2_·2H_2_O was mixed with 15 mL of deionized water, and stirred for 1 h at room temperature to obtain a clear solution. Next, 3 g of Amberlite IRA-900 resin beads were added to the solution. The mixture was stirred at room temperature for 12 h during the whole impregnation procedure. Then, the liquid was decanted and the beads were washed with deionized water and dried at 60 °C. The above-synthesized composite beads were transferred to a ceramic boat, placed in a tubular furnace, purged with nitrogen atmosphere and heated at 750, 900 °C for 4 h or 1,100 °C for 1 h at a rate of 2 °C min^−1^. It was determined that control of the temperature was critical for effective carbonization of the composite beads. The resultant material was denoted as Meso-TiO_2_/C-*x*-1, where *x* refers to the carbonization temperature. In other cases, Meso-TiO_2_/C-*x*-2 were prepared by the same procedure for Meso-TiO_2_/C-*x*-1, but using the resin derivatives before carbonization instead of starting IRA-900 resin beads in the impregnation process, that is, they were repeatedly impregnated in the solution of K_2_TiO(C_2_O_4_)_2_ with the same concentration (0.17 M). The resultant material was denoted as Meso-TiO_2_/C-750-2, Meso-TiO_2_/C-900-2 and Meso-TiO_2_/C-1100-2, respectively. For comparison, IRA-carbon sample was prepared by direct carbonization of IRA-900 resin beads at 900 °C for 4 h.

### Characterization

X-ray diffraction (XRD) patterns were measured on a Bruker D8 Advance powder diffractometer with using Cu Kα radiation (40 kV, 30 mA) for phase identification. The isotherms of nitrogen adsorption/desorption were measured at liquid nitrogen temperature using a Micromeritics TriStar 3000. The pore-size distribution was calculated using Barrett-Joyner-Halenda (BJH) model. Scanning electron microscopy (SEM) images were taken on a Hitachi S-4300 apparatus and secondary electron detector was used in the SEM measurement. Transmission electron microscopy (TEM) images were recorded on a Tecnai G^2^ instrument operating at 300 kV. For the preparation of sample for TEM analysis, the beads were ground into powders and dispersed in ethanol, and then the suspension was sonicated for 10 min. One drop of the suspension was placed on a TEM grid, and allowed to dry overnight. X-ray photoelectron spectroscopy (XPS) measurements were recorded using a PHI 5000C ESCA system with Al Kα radiation (*hν* = 1,486.6 eV). The electron take-off angle was 45º for all the samples, and the C1 *s* peak level was taken as internal reference at 284.6 eV. Elemental analyses of carbon content were performed on the elemental analyzer (Vario Micro Cube, Elementar). Thermal gravity analysis (TG) curves were monitored on a STA 449C apparatus. Ultraviolet–visible (UV/Vis) analysis was performed with a Perkin Elmer Lambda 750 spectrophotometer in matched cells.

### Photocatalysis Test

The batch photoreactor was an open to air cylindrical flask and the visible light was provided by a 300 W Xe lamp with a UV cutoff filter (λ > 420 nm). Irradiation was filtered by a circulating water cell (thickness 5.0 cm) to remove IR beams thus preventing any heating of the suspension. 30 mg of the composite beads was dispersed into 50 mL of methyl orange (MO) solution (10 mg L^−1^) under magnetic stirring at room temperature. Prior to the irradiation, the dispersion was kept in the dark for 30 min. Solutions were collected after every irradiation at certain time intervals and analyzed using a UV–vis spectrophotometer in matched quartz cells at 464 nm. For comparison, the reactions were carried out with the catalyst of P25 or IRA-carbon. The percentage of degradation was calculated as *c*/*c*_0_, where *c* is the concentration of the remaining dye solution at each irradiated time interval, while *c*_0_ means the concentration of MO solution after keeping it in the dark for 30 min in the presence of any photocatalyst. Notably, the bead shape of Meso-TiO_2_/C was unaffected during the photocatalytic tests. The beads were deposited automatically on the bottom of the vial a few seconds after stirring was stopped. Therefore, the beads could be easily separated from the reaction solution without centrifugation or filtration.

## Results and Discussion

### X-ray Diffraction Patterns

**Fig. 1:**
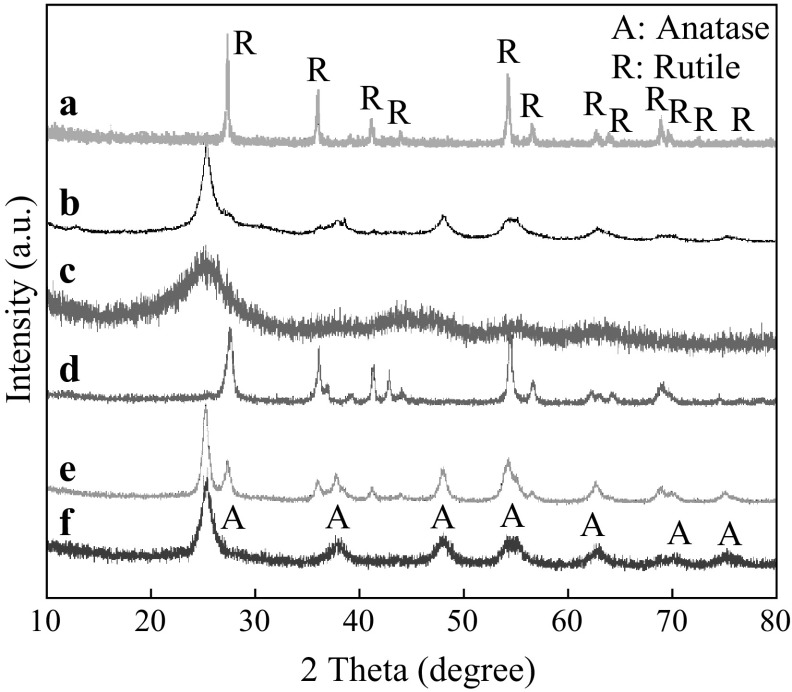
XRD patterns of *a* Meso-TiO_2_/C-1100-1, *b* Meso-TiO_2_/C-900-1, *c* Meso-TiO_2_/C-750-1, *d* Meso-TiO_2_/C-1100-2, *e* Meso-TiO_2_/C-900-2 and *f* Meso-TiO_2_/C-750-2

The crystalline structure of the TiO_2_ phase was investigated using XRD. The XRD patterns of the Meso-TiO_2_/C beads obtained at different carbonization temperatures are shown in Fig. 1. The IRA-carbon without TiO_2_ impregnation is an amorphous phase at any given carbonization temperature (not shown here). At a carbonization temperature of 750 °C, the diffraction peaks are not well defined, indicating the low crystalline TiO_2_ structural order in Meso-TiO_2_/C-750-1. When the carbonization temperature is increased up to 900 °C, the peaks assigned to anatase (JCPDS No. 21-1272) become sharp, indicating the formation of greater TiO_2_ crystallites and enhancement of crystallization, and weak peaks at 2*θ* = 27.58, 36.18, 41.38, 44.18, and 56.78 that correspond to rutile (JCPDS No. 21-1276) begin to appear in the case [33]. This observation suggests that certain amount of the anatase type may transform into the rutile type during the carbonization at 900 °C. When the carbonization temperature reaches 1,100 °C, as expected, complete transformation of anatase type to rutile is achieved. Clearly, it is found that carbonization temperature has a big influence on phase transformation of TiO_2_, which is in agreement with the results reported before [33]. The average particle size of these TiO_2_ crystallites can be estimated from the full widths at half-maximum of the (101) peak of anatase and the (110) peak of rutile by applying the Scherrer equation (Table 1), and it follows that the anatase crystallite size increases with increasing heat-treatment temperature, suggesting that high temperature leads to TiO_2_ crystal growth.

**Table 1:** Characteristics of Meso-TiO_2_/C photocatalysts prepared at different conditions

	Average particle size (nm)^a^	Mesopore size (nm)^b^	Mesopore volume (cm^3^ g^−1^)^b^	Micropore volume (cm^3^ g^−1^)^c^	Surface area (m^2^ g^−1^)	Carbon content (%)^d^
Meso-TiO_2_/C-1100-1	40.4	3.9	0.36	0.04	433	41.7
Meso-TiO_2_/C-1100-2	20.7	3.9	0.35	0.04	436	43.2
Meso-TiO_2_/C-900-1	6.0	3.8	0.28	0.07	444	50.8
Meso-TiO_2_/C-900-2	10.9	3.4; 19.0	0.38	0.05	541	40.7
Meso-TiO_2_/C-750-1	–	–	–	–	20	57.7
Meso-TiO_2_/C-750-2	6.9	–	–	–	4	46.4
IRA-carbon	–	–	0.03	0.33	741	94.6

^a^Calculated by Scherrer equation

^b^Calculated by BJH model

^c^Calculated by t-plot analysis

^d^Measured by CHN element analysis

For the bead samples of Meso-TiO_2_/C-*x*-2 prepared by repeated impregnation process, some differences are noted between diffraction patterns of Meso-TiO_2_/C-*x*-1 and Meso-TiO_2_/C-*x*-2. Meso-TiO_2_/C-750-2 possesses higher structural order than Meso-TiO_2_/C-750-1; the rutile content of Meso-TiO_2_/C-900-2 is larger than that of Meso-TiO_2_/C-900-1; weak peaks at 2*θ* = 36.7 and 42.9 assigned to TiN crystals (JCPDS No. 38-1420) are shown in Meso-TiO_2_/C-1100-2 [34]. These results suggest that repeated impregnation possibly result in enhanced exchange of negatively charged TiO(C_2_O_4_)_2_^2−^ species with anions in IRA-900.

### SEM and TEM Images

**Fig. 2:**
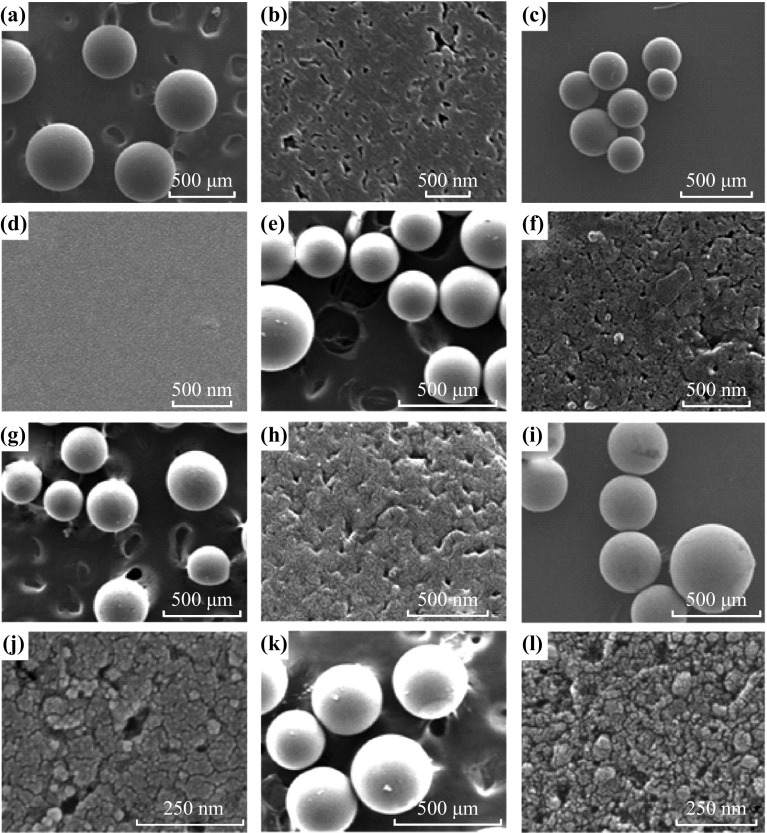
SEM images of** a**,** b** IRA-900,** c**,** d** IRA-carbon,** e**,** f** Meso-TiO_2_/C-1100-1,** g**,** h** Meso-TiO_2_/C-1100-2,** i**,** j** Meso-TiO_2_/C-900-1 and** k**,** l** Meso-TiO_2_/C-900-2

**Fig. 3:**
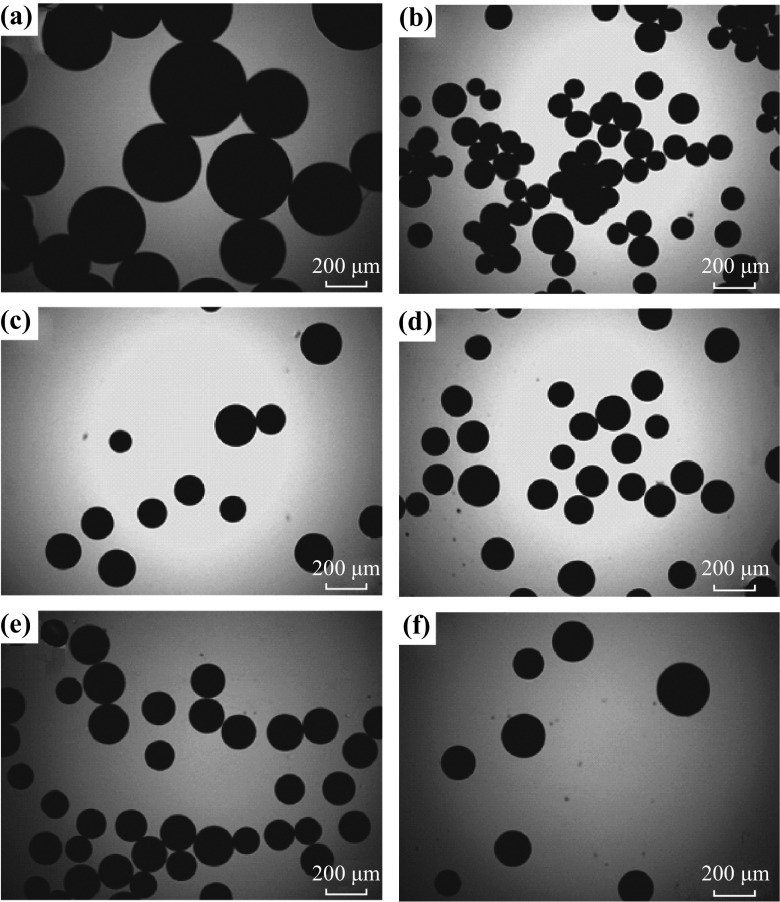
Optical images of **a** IRA-900, **b** IRA-carbon, **c** Meso-TiO_2_/C-1100-1, **d** Meso-TiO_2_/C-1100-2, **e** Meso-TiO_2_/C-900-1 and **f** Meso-TiO_2_/C-900-2

The morphology and structure of Meso-TiO_2_/C samples are clearly revealed by SEM and TEM images. After synthesis and carbonization, Meso-TiO_2_/C samples consist of hard and black beads. It has been observed that all samples contain a large amount of mesopores as well as a small quantity of macropores (Fig. 2), which might be created by the expansion of gases such as H_2_O and CO_x_ that are formed during the carbonization at high temperatures (900–1,100 °C) under nitrogen. Although spherical carbon materials are also obtained when only IRA-900 resin beads are carbonized at 900 °C under nitrogen atmosphere, the size of IRA-carbon (80–210 µm) greatly decreases compared with that of IRA-900 (290–480 µm). This clearly reveals the supporting role of TiO_2_ nanoparticles in the carbon matrix. In order to evaluate the size of these beads, the samples were characterized by optical images (Fig. 3). Based on the fact that more than fifty beads were calculated for each sample, the conclusion on the bead size of each sample was drawn in a statistical manner. Note that the size of Meso-TiO_2_/C-1100-1 (110–240 µm) and Meso-TiO_2_/C-1100-2 (115–250 µm) is smaller than that of other Meso-TiO_2_/C beads (130–240 µm for Meso-TiO_2_/C-900-1 and 130–260 µm for Meso-TiO_2_/C-900-2), which suggests that shrinkage of resin-Ti composite beads is more pronounced during the carbonization at very high temperature (1,100 °C).

**Fig. 4:**
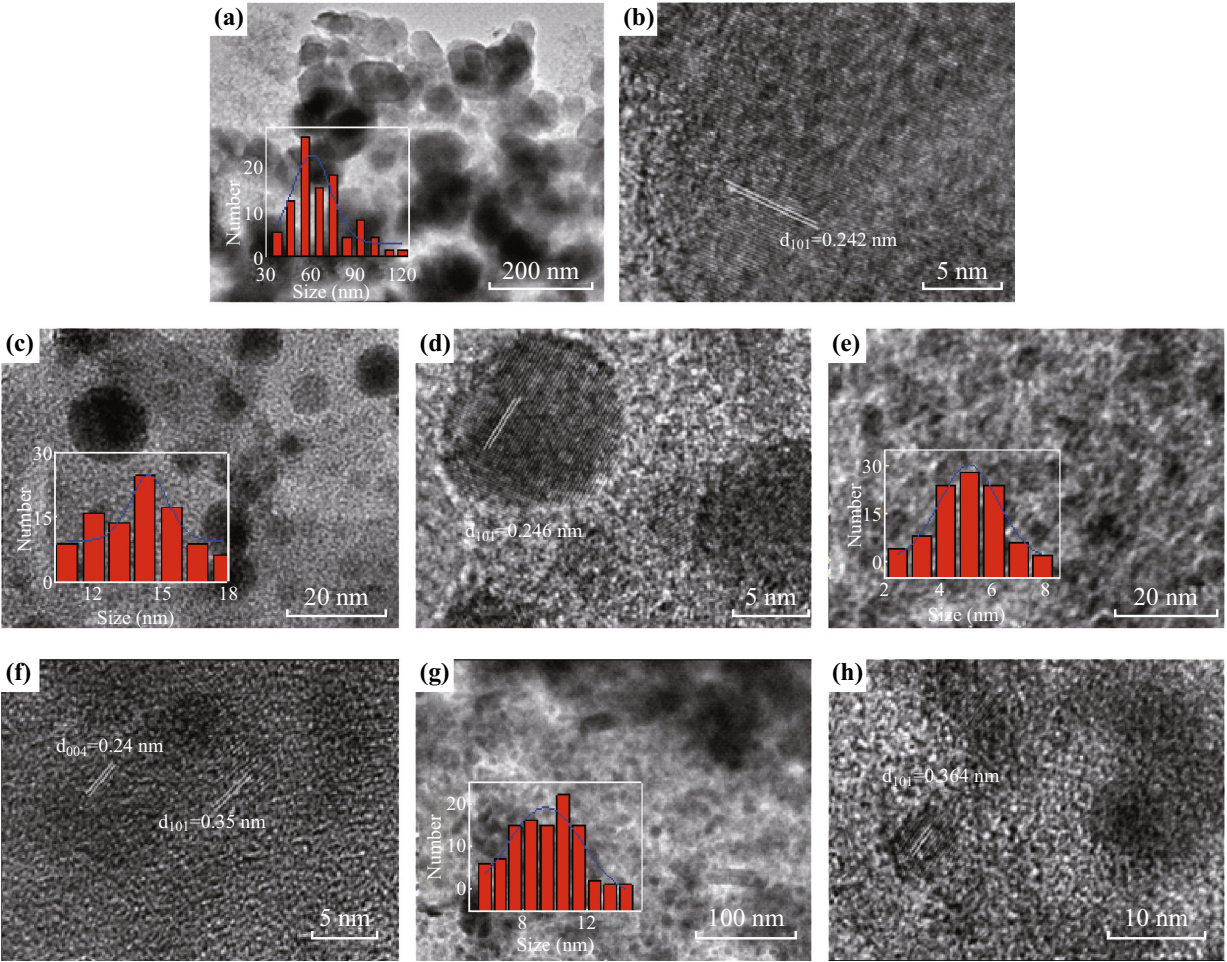
TEM images of **a**, **b** Meso-TiO_2_/C-1100-1, **c**, **d** Meso-TiO_2_/C-1100-2, **e**, **f** Meso-TiO_2_/C-900-1 and **g**, **h** Meso-TiO_2_/C-900-2. Insets are size distribution (histogram) of TiO_2_ nanoparticles in these composite beads

TEM images for Meso-TiO_2_/C beads show typical images for a composite, in which a large amount of TiO_2_ nanoparticles are well arranged in an amorphous carbon substance (Fig. 4). Based on the size distribution histogram, the size of nanoparticles in the composites follows the trend: Meso-TiO_2_/C-1100-1 > Meso-TiO_2_/C-1100-2 > Meso-TiO_2_/C-900-2 > Meso-TiO_2_/C-900-1, which is in agreement with the results obtained from XRD patterns. It seems that high carbonization temperature is favorable for larger particle size. Note here that TiO_2_ particle size in Meso-TiO_2_/C-1100-1 is much larger than that of Meso-TiO_2_/C-1100-2 even though they were carbonizated at the same condition. As seen from the optical images, the bead shrinkage in Meso-TiO_2_/C-1100-1 is more pronounced than in Meso-TiO_2_/C-1100-2. In the case, preformed nanoparticles were possibly squeezed by the shrinkage of carbon matrix and then aggregated to form larger TiO_2_ particles.

It has been also observed that disordered mesopores are clearly contained in the amorphous carbon substance, making channels for mass transport from the substance to the nanoparticles and/or large enough space for adsorption of substrates. Compared with similar results reported in mesoporous solids based titania, in which nanocrystals are randomly “glued” together by amorphous but not always mesoporous substances such as carbon, P_2_O_5_ and silica [35, 36], creation of mesopores in the carbon matrix corresponds to the utilization of the anion-exchange resin. From high-resolution transmission electron microscopy (HRTEM) images given in Fig. 4, the lattice fringe of the nanoparticles is 0.24 nm for Meso-TiO_2_/C-1100-1 and Meso-TiO_2_/C-1100-2, related to the (101) facet of rutile, and the value for Meso-TiO_2_/C-900-1 and Meso-TiO_2_/C-900-2 is about 0.36 nm, related to the (101) facet of anatase [20, 33]. It should be noted that the (004) facet of anatase with a d-spacing of 0.24 nm could also be observed in Meso-TiO_2_/C-900-1, which are rarely reported in other anatase nanoparticles [21].

### N_2_ Adsorption/Desorption Isotherms

**Fig. 5:**
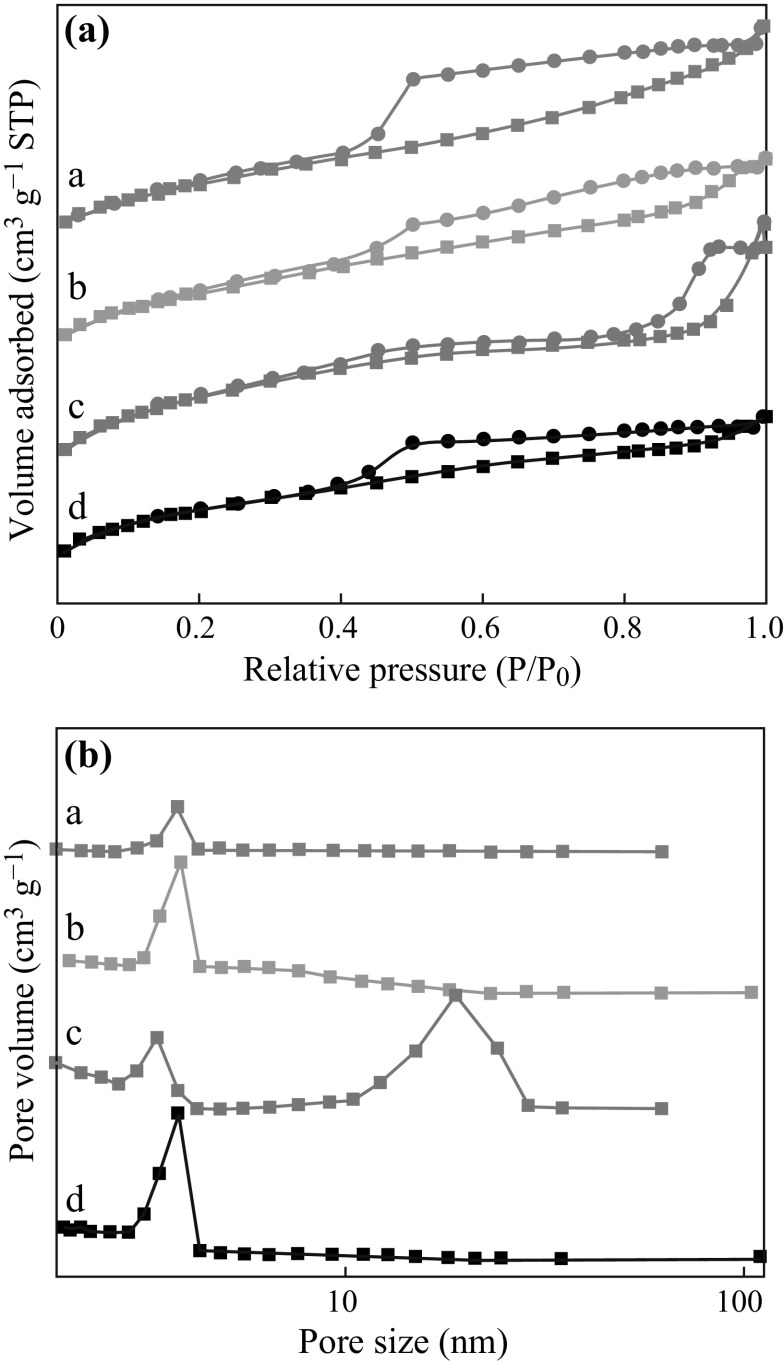
**a** Nitrogen adsorption (*filled square*)/desorption (*filled circle*) isotherms and **b** Pore-size distribution *curves* calculated from desorption branch of Meso-TiO_2_/C-1100-2 (*a*), Meso-TiO_2_/C-1100-1 (*b*), Meso-TiO_2_/C-900-2 (*c*) and Meso-TiO_2_/C-900-1 (*d*)

Figure 5 shows nitrogen adsorption/desorption isotherms for different Meso-TiO_2_/C beads. Meso-TiO_2_/C-900-1, Meso-TiO_2_/C-1100-1, and Meso-TiO_2_/C-1100-2 exhibit type IV isotherms with hysteresis loops at *P*/*P*_*0*_ > 0.4. This reveals the presence of mesopores, consistent with TEM results. The mesopores in the samples may result from the expansion of gases such as H_2_O and CO_x_ during the carbonization [37]. In contrast, the N_2_ adsorption/desorption isotherm of Meso-TiO_2_/C-900-2 has an additional hysteresis loop above *P*/*P*_*0*_ = 0.8, showing the presence of larger mesopores with a size at 18.9 nm. Combining with the TEM image of Meso-TiO_2_/C-900-2 (Fig. 4g, h), these mesopores may correspond to the void space between TiO_2_ nanoparticles and mesoporous carbon substance. Absence of such mesopores in other Meso-TiO_2_/C beads might be due to the occurrence of more pronounced shrinkage of resin-Ti composite beads during the carbonization, which took place in the cases with higher temperature (1,100 °C) and/or lower TiO_2_ content. As observed from Table 1 presenting texture properties, most of the bead samples in this work display high Brunauer–Emmet–Teller (BET) surface area and pore volume, which can favor mass transportation in the interior of the beads. It is worth noting that micropore volumes have been also estimated by performing *t*-plot analysis. Compared with their mesopore volume, the carbons are mainly mesoporous though the micropores are present in each sample. Meso-TiO_2_/C-750-1 and Meso-TiO_2_/C-750-2 give very low BET surface areas (20 and 4 m^2^ g^−1^) and no mesopores are contained in them due to the relatively low carbonization temperature.

### XPS and TG Results

**Fig. 6:**
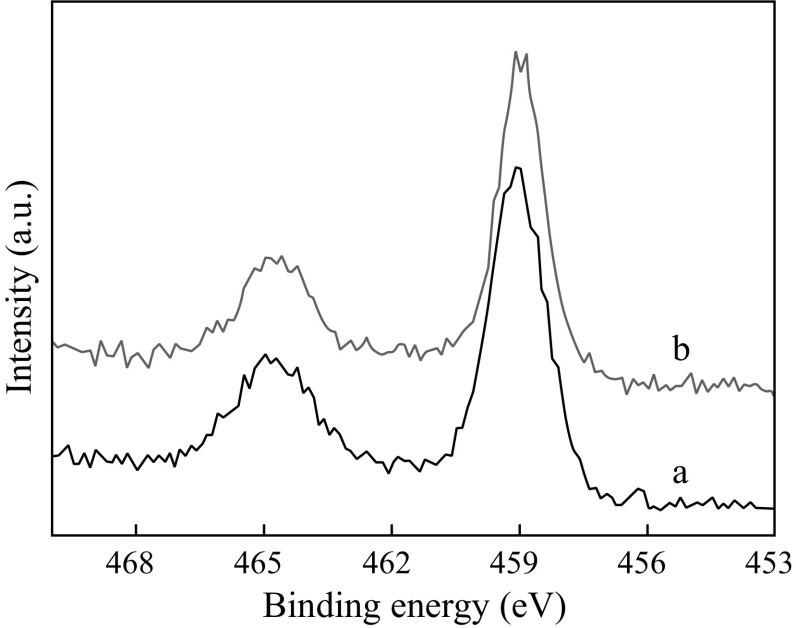
Ti 2*p* XPS spectra of *a* Meso-TiO_2_/C-900-2 and *b* Meso-TiO_2_/C-1100-1

The Meso-TiO_2_/C beads obtained via carbonization at 900 and 1,100 °C showed Ti 2*P*_3/2_ and Ti 2*P*_1/2_ XPS peaks comparable to those of bulk and neat mesoporous TiO_2_ though the signals are rather weaker in the spectra of Meso-TiO_2_/C (Fig. 6), possibly due to immobilization of TiO_2_ nanoparticles in mesopores of carbon. Notably, the spin-orbital splitting is 5.6 eV, which is slightly lower than that in bulk anatase and mesoporous titania (5.8 eV). Similar observations have been reported for nonmetal-doped anatase, and were attributed to the introduction of differently hybridized atoms into the anatase crystal lattice [20, 21, 33]. Because no signals attributed to TiC (454.9 and 460.7 eV) were detected, carbon doping in the TiO_2_ lattice is therefore responsible for the change in the spin-orbital splitting of TiO_2_. These interstitial carbon atoms have been proved to improve the degree of separation and to restrain the recombination of photo-induced electron and hole carriers in carbon-doped TiO_2_, which is conducive to improving the photocatalytic activity of the catalysts under visible-light illumination. Additionally, the interaction between carbon and TiO_2_ nanoparticles may occur during the heat treatment.

**Fig. 7:**
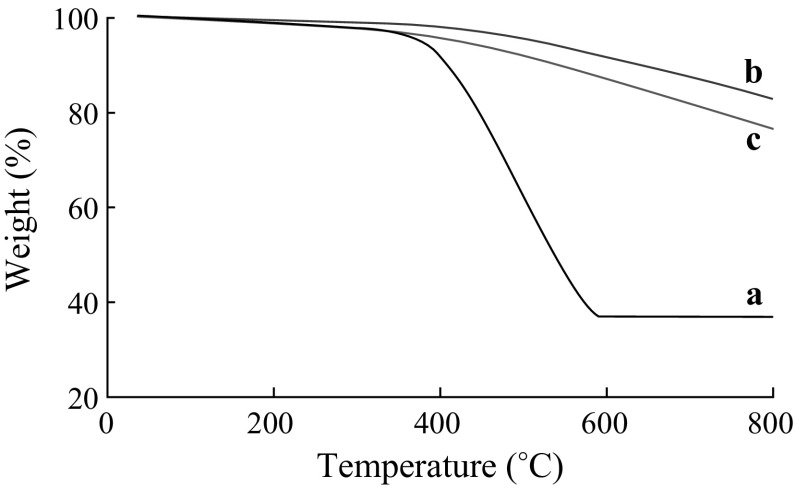
TG curves of *a* Meso-TiO_2_/C-750-1, *b* Meso-TiO_2_/C-900-1 and *c* Meso-TiO_2_/C-900-2

Figure 7 shows the TG curves of Meso-TiO_2_/C beads carried in air, which could not reach a platform value even at 800 °C, suggesting that carbonaceous materials were not burn off at high temperature. Compared with the observation that carbonaceous materials in Meso-TiO_2_/C-750-1 were burn off at around 400–600 °C, the TG results of Meso-TiO_2_/C beads prove the strong interaction between carbon and TiO_2_ nanoparticles.

**Scheme 1:**
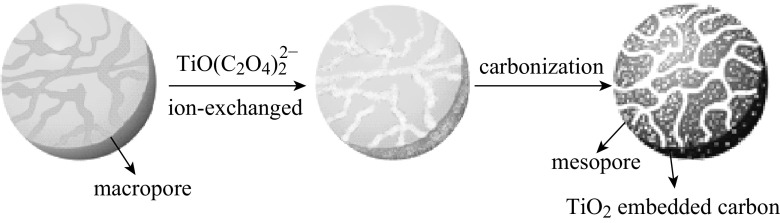
Possible schematic of the synthetic procedure for Meso-TiO_2_/C

Depending on the characterization results, we suggest a possible path for the formation of Meso-TiO_2_/C beads by the mean of anion-exchange resin beads (Scheme 1). The fabrication procedure for the bead format starts with treatment of the resin with aqueous solutions of the negatively charged TiO(C_2_O_4_)_2_^2−^ species, in which the anionic species are bound onto the host resin through electrostatic interactions. The intercondensation of TiO(C_2_O_4_)_2_^2−^ would determine the crystalline TiO_2_ nanoparticles in large mesopores and macropores of the resin during the heat treatment, which leads to the formation of composite beads of TiO_2_ nanoparticles and amorphous carbon. Simultaneously, the mesopores are generated from the gasification of volatile matter during the carbonization. Generally, the presence of mesopores in the amorphous carbon can significantly enhance adsorption capacities, especially for large adsorbates.

### Photocatalytic Activity

The visible-light photocatalytic activity of Meso-TiO_2_/C beads prepared at different carbonization temperature and impregnation process was evaluated, in which methyl orange was chosen as a degradation substrate for the adsorption and photocatalysis experiments. Before carrying out photodegradation tests, the adsorption capacity of the composite beads was tested. First, Meso-TiO_2_/C-1,100-1 (50 mg) was kept in MO solution (50 mL; 10 mg L^−1^) for 24 h and they were then separated from the solution without further washing, yielding dye-loaded Meso-TiO_2_/C-1100-1. After being cooled in air overnight, the dye-loaded Meso-TiO_2_/C-1100-1 was subjected to re-adsorption under the same concentration of MO solution. Such adsorption test was performed three times in total and each time MO molecules were almost adsorbed on Meso-TiO_2_/C-1100-1 after 24 h. This clearly indicates that such bead materials possess high adsorption capacity for MO due to their high surface areas and high pore volumes, and the saturated adsorption amount could hardly be reached under the conditions. This phenomenon also provides evidence of the high affinity between the adsorbate and the adsorbent.

**Fig. 8:**
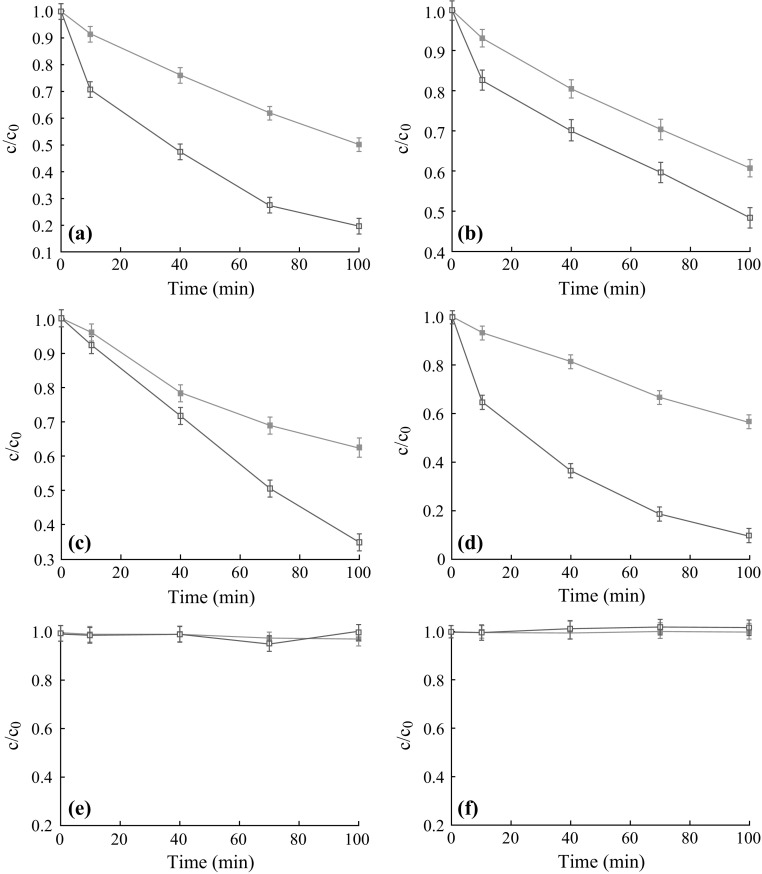
Adsorpiton (*filled square*) and degradation (*open square*) rate for methyl orange on the catalysts **a** Meso-TiO_2_/C-1100-1, **b** Meso-TiO_2_/C-1100-2, **c** Meso-TiO_2_/C-900-1, **d** Meso-TiO_2_/C-900-2, **e** Meso-TiO_2_/C-750-1 and **f** Meso-TiO_2_/C-750-2 as a function of the contacting time

**Fig. 9:**
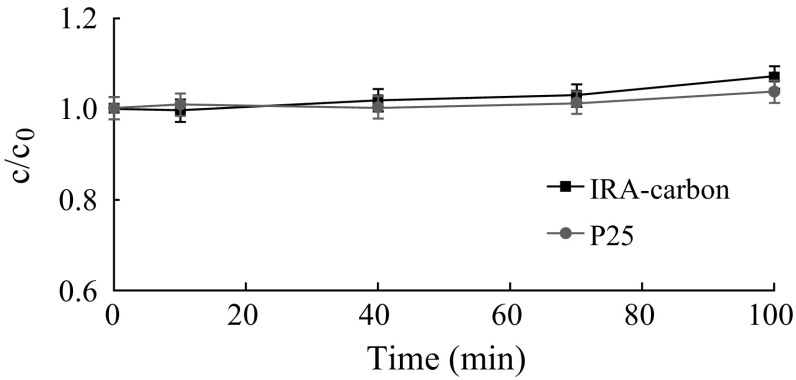
Degradation rate for methyl orange on P25 and IRA-carbon as a function of the contacting time

To understand the nature of the catalytic center, we performed series of adsorption and catalysis tests for the degradation of the azo dye, which consisted of 100 min for adsorption in the dark and for illumination under visible light (Fig. 8). A blank experiment was carried out with the MO solution, without any photocatalytic material, proving that there is no direct photolysis of the dye molecules. Compared with their adsorption capacity, as observed from Fig. 8, the Meso-TiO_2_/C samples carbonizated at 900 and 1,100 °C have high visible-light photocatalytic activity. Prior to the irradiation, the dispersion was kept in the dark for 30 min. About 21, 36, 27, and 23 % of the dye calculated by *c*/*c*_0_ was adsorbed on Meso-TiO_2_/C-900-1, Meso-TiO_2_/C-900-2, Meso-TiO_2_/C-1100-1, and Meso-TiO_2_/C-1100-2, respectively. With the extension of the illumination time, the degradation efficiency dramatically increased, reflecting degradation of the residue dye in the mesopores of Meso-TiO_2_/C. After 100 min of illumination time, about 50–90 % of the dye was eliminated due to the simultaneous adsorption and photocatalysis. These observations illustrate that the dyes adsorbed in mesoporous carbon-titania composite beads can be degraded by crystalline TiO_2_ nanoparticles inside carbon pore walls under visible light. Once the dyes in pores are mineralized, the occupied pores are empty, thus, enough space can be provided again for re-adsorption. These features may be attributed to the synergetic effects of the factors in terms of mesopores, the doping of carbon element into TiO_2_ lattice and interaction between the carbon and TiO_2_ [20, 34]. The mesopores in the interior favor the absorption of MO and simultaneously cause the MO molecules close to the TiO_2_ nanoparticles. The carbon doping increases the visible-light absorption. This implies that Meso-TiO_2_/C beads can be activated by visible light and more photo-generated electrons and holes can be created and participate in the photocatalytic reactions. Some other parallel experiments were also carried out on P25 and IRA-carbon (Fig. 9). Combined with the result that Meso-TiO_2_/C-750-1 and Meso-TiO_2_/C-750-2 exhibited low adsorption capacity and photocatalytic activity, proving that the mesopores in the beads play an important role in high adsorption capacity and that the doping of C element and/or interaction between mesoporous carbon and crystalline titania nanoparticles is vital for visible-light-induced degradation of the azo dye methyl orange. The photocatalytic activity (at reaction time of 100 min) of these composites follows the trend: Meso-TiO_2_/C-900-2 > Meso-TiO_2_/C-1100-1 > Meso-TiO_2_/C-900-1 > Meso-TiO_2_/C-1100-2 >> Meso-TiO_2_/C-750-2 and Meso-TiO_2_/C-750-1 (Fig. 8), indicating that the TiO_2_ content, mesopore volume and the interaction between TiO_2_ nanoparticles and carbon have significant influence on the photocatalytic activity and that anatase and rutile are both active for dye degradation under visible light.

Some researchers reported that photocatalytic reaction occurred under irradiation with visible light with active carbon samples and systems of condensed benzene rings in active carbon play the role of the organic semiconductor molecules [38]. However, in our case, IRA-carbon did not exhibit photocatalytic activity of MO under visible light. It is clear that the visible-light activity of the Meso-TiO_2_/C beads should be described by taking into account other parameters. An explanation lies on the carbon doping into the TiO_2_ lattice during the formation of the composite beads, which is expected to favor lowering the bandgap energy of Meso-TiO_2_/C composites. Consequently, significant enhancement in the photoactivity under visible light was obtained, as the researches described elsewhere [20, 33]. The carbonaceous species formed by doped C atoms acted as a photosensitizer, which can be excited and inject electrons into the conduction band of TiO_2_, then the electron is transferred to the molecular oxygen adsorbed in the mesopores of Meso-TiO_2_/C, producing ·O_2_^−^ and ·OH radicals that are capable of degrading MO [39]. An additional reason for the high visible-light activity of the Meso-TiO_2_/C beads may be attributed to the strong interaction between carbon and TiO_2_ nanoparticles [20, 21, 33]. It is well known that amorphous carbonaceous material is a kind of strongly dye-absorbing carbons. In the present work, methyl orange adsorbed in the mesopores of the Meso-TiO_2_/C beads can absorb visible light and is excited, which is similar as the results reported elsewhere [20, 21, 33]. In addition, the enriched and excited dye molecules in the mesopores can fully access active crystalline titania nanoparticles via the present mesopore channels. It has been proved that the conduction band of TiO_2_ plays an important role and the appropriate corresponding potential enables the excited dye molecules to inject electrons into the conduction band of a TiO_2_ particle [39]. It is supposed, thus, carbon doping modifies the bandgap of crystalline TiO_2_ nanoparticles, and may facilitate the above electron-injection process, yielding dye radicals. These dye radicals are transient, active species and decompose into small molecules and ever further to CO_2_ by complicated reactions involving oxygen.

**Fig. 10:**
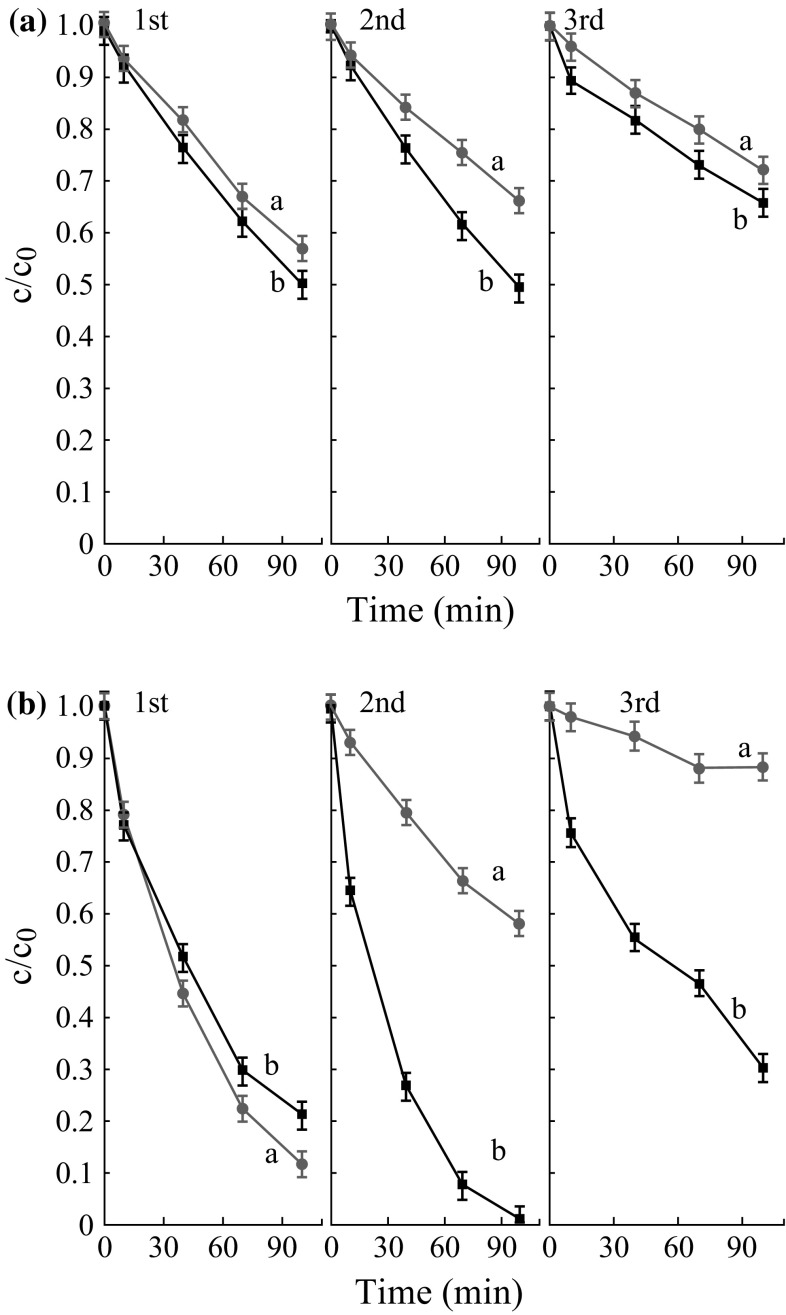
Recycling runs in **a** adsorption and **b** photodegradation of methyl orange over Meso-TiO_2_/C-900-2 and Meso-TiO_2_/C-1100-1

**Fig. 11:**
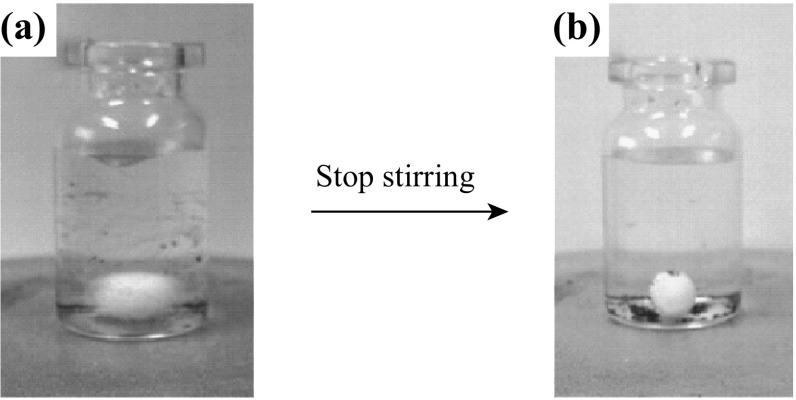
Meso-TiO_2_/C-1100-1 **a** under stirring and **b** in static conditions (a few seconds after stopping the stirring)

In view of potential industrial applications, recyclability is an important parameter in evaluating a catalyst. It has been well known that TiO_2_ nanoparticles are hard to separate from the aqueous or gaseous pollutants and aggregate easily in suspension, which would significantly affect the activity and stability of the photocatalyst. To complete the evaluation of our novel catalyst Meso-TiO_2_/C beads, the materials were recovered from the reaction mixture and reused in several photocatalytic cycles (Fig. 10). The straightforward recycling procedure for Meso-TiO_2_/C beads only required washings in ethanol at room temperature without any calcination. Note that the bead shape of Meso-TiO_2_/C was unaffected by stirring during the catalytic tests. The beads were deposited automatically on the bottom of the vial a few seconds after stirring stopped (Fig. 11). Therefore, the beads could be easily separated from the reaction solution without centrifugation or filtration. The catalytic activity of Meso-TiO_2_/C-1100-1 remained at 85 % of the first run after three cycles, indicative of the good stability of the Meso-TiO_2_/C beads catalyst. Interestingly, in the second run, reused Meso-TiO_2_/C-1100-1 surprisingly displayed higher photocatalytic activity than the fresh one, which is possibly because more facets of TiO_2_ particles are exposed during stirring in the photocatalysis test. The reused Meso-TiO_2_/C-1100-1 was characterized by TEM and no big difference on TiO_2_ particles was observed compared with those in the fresh sample. Therefore, further investigation for its reusability is still in progress.

## Conclusion

Mesoporous TiO_2_/C composite beads have been prepared using anion-exchange resin as carbon source and applied as an adsorbent–photocatalyst to eliminate methyl orange under visible light. Dispersed TiO_2_ nanoparticles were confined by mesoporous carbon pore walls, which prevented their aggregation during heating, and served to modify their surface and electronic structure. The composites showed a synergy effect by carbon and TiO_2_ nanoparticles on elimination of the dye from water, that is, large mesopore size and high surface area facilitate the adsorption of the dye and well-dispersed TiO_2_ nanopartilces are active to degrade dye molecules through the interface between carbon and TiO_2_ nanoparticles. Also, mesopores allowed full access of the dye molecules to the surface of TiO_2_ nanoparticles, where they were degraded. As a result of these unique features, the composite showed both high adsorption capacity and visible-light-induced photocatalytic activity. The doping of carbon into the TiO_2_ lattice and strong interaction between carbon and TiO_2_ nanoparticles has been proved to improve the photodegradation performances toward dyes. Notably, these mesoporous TiO_2_/C composites could be easily separated from the dye solution due to their bead format, which is more attractive for industrial treatment of organic contaminants.
